# Successful Midwifery

**Published:** 1884-02

**Authors:** 


### Successful Midwifery.

The most successful results ever obtained in the Maternite Hospital, Paris (a mortality of only ¾ of one per cent.), have been reached in the new pavilion, of which M. Tarnier says: "Each patient there has a separate room, entered from without, so that a nurse can only pass from one to another by going outside into the open air. The furniture is of japanned iron; the floors, walks and ceilings of impermeable concrete. The mattresses and pillows are stuffed with cut chaff, which is burnt after use in every single case. For the McIntosh sheet is substituted one of brown paper, made impermeable by pitch—this is burnt after use. For the washing of the genitals, weak solutions of bichloride of mercury are employed as being the best and most powerful germicide."—*Obst. Gaz.*

